# Feasibility of Using Ceftazidime-Avibactam as a Therapeutic Option for Bloodstream Infections Caused by Multidrug-Resistant Enterobacterales and Pseudomonas aeruginosa Based on Its Susceptibility Profile

**DOI:** 10.7759/cureus.37002

**Published:** 2023-04-01

**Authors:** Ketan Priyadarshi, Sarumathi Dhandapani, Monika Sivaradjy, Lakshmi Shanmugam, Apurba S Sastry

**Affiliations:** 1 Department of Microbiology, All India Institute of Medical Sciences, Patna, IND; 2 Department of Microbiology, Jawaharlal Institute of Postgraduate Medical Education and Research, Puducherry, IND; 3 Department of Microbiology, Employees' State Insurance Corporation Medical College and Post Graduate Institute of Medical Science and Research, Chennai, IND

**Keywords:** bloodstream infections, carbapenem resistant organisms(cro), ceftazidime avibactam, gram negative bacteremia, multi-drug resistant organisms (mdro), therapeutic choice

## Abstract

Background

In the era of increased antimicrobial resistance, there are limited therapeutic options available for the treatment of bacteremia caused by multidrug-resistant organisms (MDROs). This study aims to find out the feasibility of using ceftazidime/avibactam (CZA) as a therapeutic option for bloodstream infections caused by multidrug-resistant (MDR) Enterobacterales and *Pseudomonas aeruginosa* based on its susceptibility profile.

Materials and methods

The isolates were routinely subjected to antimicrobial susceptibility testing (AST) by an automated AST system (VITEK-2). Those isolates found as MDR (resistant to at least one drug for ≥3 antimicrobial classes) were tested against CZA by Kirby-Bauer’s disk diffusion (kb-DD) method.

Results

A total number of 293 MDR Enterobacterales and 31 MDR *P. aeruginosa* isolates were included. Of these, 87.3% of isolates were found as carbapenem-resistant (CR), whereas 12.7% of isolates were found as carbapenem susceptible. About 30.6% of MDROs were susceptible to CZA. Among carbapenem-resistant organisms (CROs), CR *Klebsiella pneumoniae*(33.5%) is most susceptible to CZA, compared to CR *P. aeruginosa*(0%)and CR*Escherichia coli*(3.2%). Among the MDR isolates that were susceptible to CZA (30.6%), the majority had poor susceptibility against other β-lactam-β-lactamase inhibitor (BL-BLI) agents. Among all antimicrobial agents tested against CROs, colistin (96%) was found to have the best susceptibility profile.

Conclusion

It is observed that CZA is an acceptable therapeutic option for the treatment of bacteremia caused by MDROs, especially CROs. Therefore, it is important for the laboratories to perform the AST for CZA if the healthcare settings intend to use CZA for the management of such “difficult-to-treat” bloodstream infections.

## Introduction

Antimicrobial resistance (AMR) has emerged as a rising global threat over the past few decades. The spread of multidrug-resistant organisms (MDROs) fueled by antibiotic overuse in humans, animals, and the environment, as well as a paucity of new antibiotic development, has led to “difficult-to-treat” infections with increased morbidity and mortality [1,2]. There had been a few initiatives for combating AMR and improving new antibiotic development, namely “bad bugs, no drugs” [2,3] and the 10 x '20 Initiative [2,4,5]. There is an urgent need for the development of new antimicrobial agents to treat MDRO infections, particularly those caused by carbapenem-resistant organisms (CROs), which are also identified as WHO priority pathogen list [6].

There are limited therapeutic options currently available for the treatment of MDR infections, among which noteworthy to mention are ceftolozane/tazobactam and ceftazidime/avibactam (CZA), which are novel β-lactam/β-lactamase inhibitor combination antibiotics. Avibactam is a novel non-β-lactam β-lactamase inhibitor that binds reversibly to β-lactamase and thus inactivates multiple β-lactamase molecules simultaneously [1].

In addition to its activity against extended-spectrum β-lactamase (ESBL) producing Gram-negative bacilli (GNBs), CZA also has acceptable efficacy against Ambler class A and C β-lactamases and some Ambler class D β-lactamases producing organisms viz. AmpC β-lactamase producing *Pseudomonas aeruginosa*, Enterobacterales, and even CROs, which belong to *Klebsiella pneumoniae* carbapenemase (KPC) and OXA-48-like family [1,7,8]. The only limitation of CZA is its reduced efficacy over metallo-β-lactamase (MBL) producing GNBs [1]. CZA was approved by the US Food and Drug Administration (USFDA) in 2015 for the treatment of complicated intra-abdominal infections and complicated urinary tract infections (cUTI), including pyelonephritis [9]. However, its use can be extended to treat other site-specific infections, including bloodstream infections (BSIs) [10].

In resource-limited countries like India, the availability of CZA as a therapeutic choice is very limited, often rendering the use of colistin and tigecycline or minocycline as therapeutic options for the management of MDROs, especially CROs, and “difficult-to-treat infections.” In addition, the availability of antimicrobial susceptibility testing (AST) data of CZA against MDROs is lacking in resource-limited countries like India [11]. More so, the usage of CZA should be reserved only for serious, life-threatening infections like BSIs, where limited AST-guided treatment options are available. To maintain its effectiveness and prevent the emergence of AMR, the therapeutic use of CZA should always be AST-guided, not empirical. All the above-mentioned reasons necessitate the laboratory to perform the susceptibility testing of CZA against MDROs, especially CROs, in those healthcare settings that are planning to use CZA as a therapeutic option for the management of “difficult-to-treat” BSIs.

## Materials and methods

The present study was a prospective, observational study conducted in a tertiary care hospital in southern India from October 2020 to March 2021. The blood culture specimens from cases of suspected sepsis were collected in the BacT/ALERT automated blood culture bottles by the treating clinicians. These bottles, which were sent to the microbiology laboratory during this period, were included in the study.

As a part of the standard protocol of the laboratory, the blood cultures upon receipt were immediately loaded in the VIRTUO BacT/ALERT (bioMérieux, France) system and incubated aerobically at 37°C for five days. The positively flagged blood culture bottles that were unloaded from the instrument were subjected to gram staining and subcultured on 5% sheep blood agar (SBA), chocolate agar (CA), and MacConkey agar (MAC). Colonies were identified by matrix-assisted laser desorption ionization-time of flight mass spectrometry (MALDI TOF-MS) (VITEK-2 MS, bioMérieux, France). Subsequently, AST was performed by the broth microdilution method using VITEK-2 automated system, and the minimum inhibitory concentration (MIC) obtained was interpreted using clinical breakpoints in the Clinical and Laboratory Standards Institute (CLSI) M-100 document [12]. The following antimicrobials were tested by VITEK-2-amoxicillin-clavulanate (AMC), gentamicin, ciprofloxacin, ceftriaxone, ceftazidime, cotrimoxazole, cefepime, piperacillin-tazobactam (PTZ), cefoperazone-sulbactam (CFS), amikacin, meropenem, imipenem, ertapenem, tigecycline, and colistin.

AST of CZA was performed by Kirby-Bauer’s disk diffusion (kb-DD) method using CZA 30/20 µg disk (BD BBL/Oxoid, bioMérieux, France). The isolates chosen for CZA testing were MDR Enterobacterales and *P. aeruginosa*, defined as nonsusceptible/resistant to at least one member of three or more different classes of antimicrobials tested [13]. All other organisms were excluded from the study as there are no clinical breakpoints for CZA available in the CLSI M-100 document [12]. The MDR Enterobacterales and *P. aeruginosa* isolates were further classified into the CRO, defined as resistant to either meropenem or imipenem or ertapenem, and carbapenem-susceptible organism (CSO), defined as susceptible to all carbapenems tested. The results of CZA testing by kb-DD were interpreted as per the CLSI M-100 document [12]; isolates with zone diameters of ≥21 mm and ≤20 mm were considered susceptible and resistant, respectively, for both Enterobacterales and *P. aeruginosa*.

## Results

A total number of 324 MDRO isolates (defined as resistance to at least one member of three or more different classes of antimicrobials tested) obtained from culture-proven BSIs were included in the study. Of these, 87.3% (283) isolates were CR, i.e., resistant to either meropenem or imipenem or ertapenem, and 12.7% (41) isolates were carbapenem susceptible. The organism group-wise distribution of MDR Enterobacterales (which includes *E. coli, K. pneumoniae, Enterobacter cloacae, Providencia *species*, Citrobacter braakii, and Proteus mirabilis*) and *P. aeruginosa* is depicted in Table 1. Enterobacterales accounted for 90.4% of isolates, of which *K. pneumoniae* (57.1%) and *E. coli* (26.9%) were the major isolates.

**Table 1:** MDR Enterobacterales and Pseudomonas aeruginosa included in the study. MDROs: multidrug-resistant organisms; CRO: carbapenem-resistant organisms; CSO: carbapenem-susceptible organisms. *Others, includes *Providencia* (1), *Citrobacter braakii* (1), and *Proteus mirabilis* (3).

MDROs	CRO (% (n/N)) (N = 283)	CSO (% (n/N)) (N = 41)	Total (% (n/N)) (N = 324)
Enterobacterales	89.4 % (253/283)	97.6 % (40/41)	90.4% (293/324)
Escherichia coli	22.3 % (63/283)	58.5% (24/41)	26.9 % (87/324)
Klebsiella pneumoniae	61.1 % (173/283)	29.3 % (12/41)	57.1 % (185/324)
Enterobacter cloacae	4.2% (12/283)	9.8% (4/41)	4.9% (16/324)
Others*	1.8 % (5/283)	0 (0/41)	1.5 % (5/324)
Pseudomonas aeruginosa	10.6% (30/283)	2.4 % (1/41)	9.6 % (31/324)

The susceptibility profile of MDR Enterobacterales and *P. aeruginosa* for CZA is shown in Table 2. About 30.6% of MDROs were susceptible to CZA, whereas the CZA susceptibility among CRO and CSO was 21.6% and 92.7%, respectively. Among CRO, CR *K. pneumoniae* (33.5%) is most susceptible to CZA, compared to CR *P. aeruginosa* (0%) and CR *E. coli* (3.2%).

**Table 2:** Susceptibility profile of MDR Enterobacterales and Pseudomonas aeruginosa for CZA. CZA: ceftazidime/avibactam; CRO: carbapenem-resistant organisms; CSO: carbapenem-susceptible organisms; MDR: multidrug-resistant.

Organisms	CZA susceptibility among CRO (%(n/N))	CZA susceptibility among CSO (%(n/N))	CZA susceptibility among MDR Isolates (%(n/N))
Enterobacterales	24.1% (61 /253)	92.5% (37/40)	33.4% (98/293)
Escherichia coli	3.2% (2 /63)	91.7% (22/24)	27.5% (24/87)
Klebsiella pneumoniae	33.5% (58/173)	91.7% (11/12)	37.3% (69/185)
Enterobacter cloacae	8.3% (1 /12)	100% (4/4)	31.2% (5/16)
Others	0% (0 /5)	0 (0/0)	0% (0/5)
Pseudomonas aeruginosa	0% (0/30)	100% (1/1)	3.2% (1/31)
Total	21.6% (61/283)	92.7% (38/41)	30.6% (99/324)

Table 3 depicts the susceptibility profile of CZA when compared to other β-lactam-β-lactamase inhibitor (BL-BLI) antimicrobials such as AMC, PTZ, and CFS among MDR Enterobacterales and *P. aeruginosa*. Among the MDRO isolates that were susceptible to CZA (30.6%), the majority had poor susceptibility to other BL-BLI agents, namely AMC, PTZ, and CFS (2.9%, 6.2%, and 8.6%, respectively). On the contrary, of the MDRO isolates resistant to CZA (69.4%), only 1.1%, 0.6%, and 0.6% showed additional susceptibility to other BL-BLI agents, namely AMC, PTZ, and CFS, respectively.

**Table 3:** Susceptibility profile of CZA compared to other BL-BLIs among MDR Enterobacterales and Pseudomonas aeruginosa. CZA, ceftazidime-avibactam; AMC, amoxicillin-clavulanate; PTZ, piperacillin-tazobactam; CFS, cefoperazone-sulbactam; BL-BLIs, β-lactam-β-lactamase inhibitors; MDR: multidrug-resistant.

CZA	AMC susceptibility	PTZ susceptibility	CFS susceptibility
CZA (S) (30.6%)	2.9 %	6.2%	8.6%
CZA (R) (69.4%)	1.1%	0.6%	0.6%

The susceptibility profile of all major antimicrobial agents tested was evaluated as compared with CZA for CR MDR isolates in Table 4 to find out the available therapeutic options of these agents to treat MDR CROs. Among all CROs tested, colistin (96%) has been found to have the best susceptibility profile, while tigecycline (29.4%) and aminoglycosides (18.4%) were comparable to CZA (21.6%). Cotrimoxazole (9.5%), ciprofloxacin (0.7%), and other BL-BLI agents, namely CFS (0.35%), PTZ (0.35%), and AMC (1.7%), have poor susceptibility profiles.

**Table 4:** Therapeutic options for CR MDROs against other antimicrobials based on their susceptibility profiles. CZA: ceftazidime-avibactam; CIP: ciprofloxacin; COT: cotrimoxazole; AMC: amoxicillin-clavulanate; PTZ: piperacillin-tazobactam; CFS: cefoperazone-sulbactam; AMG: aminoglycosides; TGC: tigecycline; COL: colistin; CR: carbapenem-resistant; MDROs: multidrug-resistant organisms.

Organisms	CZA	CIP	COT	AMC	PTZ	CFS	AMG	TGC	COL
Total CRO	21.6%	0.7%	9.5%	1.7%	0.4 %	0.4%	18.4%	29.4%	96.0%
CR Enterobacterales	24.1%	0.8%	9.5%	1.7%	0.4%	0.4%	20.2%	29.4%	96.0%
Escherichia coli	3.2%	6.3%	0	0	3.2%	0	33.3%	81%	98.4%
Klebsiella pneumoniae	33.5 %	9.9%	2.3%	0	33.5%	0.6%	16.3%	11.6%	94.8%
Enterobacter cloacae	8.3%	16.7%	IR	0	8.3%	0	16.7%	16.7%	100%
Others	0	20%	0	20%	0	0	20%	0	100%
CR *Pseudomonas aeruginosa*	0	0	IR	IR	0	0	3.3%	IR	96.7%

Among CR Enterobacterales (CRE), the antimicrobials with better susceptibility profiles are colistin (96%), tigecycline (29.4%), and aminoglycosides (20.2%) as compared to CZA (24.1%). CZA proved particularly better in cases of CR *K. pneumoniae* with a susceptibility profile of 33.5%, as compared with tigecycline (11.6%) and aminoglycosides (16.3%). CZA appeared to have poor susceptibility (3.2%) for CR *E. coli*, for which tigecycline (81%) and aminoglycosides (33.3%) flared much better. Among CR *P. aeruginosa*, none of the antimicrobials, except colistin (96.7%) and aminoglycosides (3.3%), were found to be susceptible.

## Discussion

There has been an increased surge in the prevalence of “difficult-to-treat” BSIs due to MDROs worldwide. One important group of MDRO is CROs, which are resistant to various β-lactams, including carbapenems, leaving with limited therapeutic options [10, 14]. The older generation BL-BLI combination classes of antimicrobials, namely AMC, ampicillin-sulbactam, PTZ, ticarcillin-clavulanate, CFS, etc., are almost ineffective for these CROs. A series of novel BL-BLIs has been introduced to strengthen the therapeutic options against these bugs. These are ceftolozane-tazobactam, imipenem-relebactam, meropenem-vaborbactam, and the antimicrobial evaluated in the present study, "ceftazidime-avibactam" [15].

As per the Infectious Diseases Society of America (IDSA), CZA can be considered as a preferred treatment option as monotherapy for infections caused by CRE, especially for carbapenemase-negative, KPC β-lactamase, and OXA-48-like β-lactamase-producing CRE infections along with “difficult-to-treat” resistance (DTR) *P. aeruginosa*, and in combination with aztreonam for New Delhi MBL (NDM) and other MBL-producing CRE infections. Combination antibiotic therapy (i.e., the use of a β-lactam agent in combination with other agents such as an aminoglycoside, fluoroquinolone, or polymyxin) is not routinely recommended for the treatment of infections caused by CRE [10].

In the present study, we tried to evaluate the susceptibility profile of CZA against MDR Enterobacterales and MDR *P. aeruginosa*. We separately analyzed the CZA susceptibility profile among CROs and CSOs. The purpose of this study was to find out the susceptibility profile of CZA in our local settings and to determine if it can be considered as a therapeutic option for MDROs. CLSI mentions CZA as a group-B antimicrobial, defined as the drugs that are used for routine primary testing but may be reported selectively [12]. However, in the Indian setting, CZA should be used as a RESERVE class of antimicrobials, used only for MDRO susceptible to CZA and with limited treatment options, as advocated by WHO [14, 16]. Therefore, we limited this study only to MDROs.

In our locations, the majority of the MDROs causing BSIs to belong to Enterobacterales (90.4%), in which *K. pneumoniae* (57.1%) and *E. coli* (26.9%) were the most common organisms, followed by *P. aeruginosa* (9.6%), similar to Leal et al. [17] and Diekema et al. [18]. The majority of *K. pneumoniae* (93.5%; 173/185) were CROs, while almost all of *P. aeruginosa* (96.8%; 30/31) were CROs. We attempted to analyze the susceptibility profile of CZA for CRO and CSO separately. We observed a contrasting difference in the susceptibility profile of CZA among these two MDRO groups. While the CSOs were almost susceptible to CZA (92.7%), the susceptibility among CROs for CZA was low (21.6%), comparable to a few studies that also found susceptibility as low as 45% [19]. Among CROs, CR *K. pneumoniae* (33.5%) is more susceptible to CZA, compared to CR *P. aeruginosa* (0%) and CR *E. coli* (3.2%). This indicates that CZA may be considered as a better therapeutic option in BSIs caused by CR *K. pneumoniae* as compared to other CROs.

The susceptibility profile of CZA was compared with that of old-generation BL-BLIs, and it was found that CZA is far more susceptible than old-generation BL-BLIs, namely CFS, PTZ, and AMC when tested against MDROs (30.6% vs. 8.6%, 6.2%, 2.9%, respectively) and also against CROs (21.6% vs. 0.4%, 0.4%, 1.7%, respectively). Therefore, CZA may be reserved for therapy for infections caused by MDROs resistant to old-generation BL-BLIs.

We compared the susceptibility profile of CZA with that of other antimicrobials that can be used as therapeutic options for the treatment of CROs. The susceptibility profile of CZA for CRE was comparable to that of aminoglycosides and tigecycline; however, it grossly varied among CR *K. pneumoniae* and CR *E. coli*. For CR *K. pneumoniae*, CZA was far superior to any other therapeutic options; while for CR *E. coli*, it was observed that tigecycline and aminoglycosides were superior in vitro to CZA. In our study, colistin had a much higher in vitro susceptibility to CRE than CZA (96.0% vs. 24.1%).

But aminoglycosides and colistin are both nephrotoxic. Tigecycline blood levels are low relative to MIC and not considered as a good choice for BSIs, and its use is associated with increased mortality rates [20]. IDSA advocates against the usage of polymyxin B and colistin for the treatment of infections caused by CRE, except for uncomplicated CRE cystitis. [10] When comparing the patient outcome treated with CZA versus colistin, van Duin et al. reported that CZA has a better all-cause 30-day hospital mortality than colistin (9% versus 32%) [21]. Therefore, CZA can still be regarded as a valuable choice as compared to aminoglycosides, tigecycline, and colistin for the treatment of CRE bacteremia.

In our study, CZA was far inferior as a therapeutic option for CR *P. aeruginosa*, i.e., all the isolates were resistant to CZA, in contrast to various other studies in which resistance to CZA among CR *P. aeruginosa* varied from 14% to 33% [22, 23]. The reason for this finding could be because CZA is ineffective against MBL-producing GNBs, which is one of the most common and clinically significant mechanisms of carbapenem resistance in *P. aeruginosa* [24], although characterization of the type of β-lactamase genes was not performed in the present study. In context to the terminologies depicting resistance, the concept of DTR was recently proposed and defined as non-susceptibility to all first-line agents, namely all β-lactams, including carbapenems and BL-BLI combinations, and fluoroquinolones, e.g., for *P. aeruginosa*, resistance to PTZ, ceftazidime, cefepime, aztreonam, meropenem, imipenem-cilastatin, ciprofloxacin, and levofloxacin [10, 20]. In our study, all the CR *P. aeruginosa* isolates were also found to be DTR. Therefore, in our setting, CZA was found to be an ineffective therapeutic option for the treatment of "difficult-to-treat" *P. aeruginosa*.

Limitations of the study

Treatment with CZA monotherapy is justified only for KPC β-lactamase and OXA-48-like β-lactamase-producing organisms, and for treating MBL-producing organisms, CZA needs to be combined with aztreonam. Additional testing is required to demonstrate the expression of those enzymes for directing the antimicrobial therapy toward monotherapy or combinational therapy. The main limitation of this study is that we did not perform laboratory tests to detect these enzymes, which would further rationalize the antimicrobial therapy. The study did not include the objective of correlating the clinical response of the patient who was started with CZA, which is another limitation of the study.

## Conclusions

To conclude, CZA is a valuable therapeutic option for the management of CR Gram-negative BSIs. The susceptibility testing should routinely be performed in settings where the prevalence of MDROs, including CRE and CR *P. aeruginosa, *is high. Also, the availability of this antimicrobial in hospitals in resource-limited countries is still a big problem, but it should be made readily available for usage. In the present study, CZA looks like a promising option for the management of CRE, especially CR *K. pneumonia *BSIs for usage at our facility. Further, the clinical response of CZA therapy in patients with sepsis can be analyzed in future studies.
