# Correction: Feasibility of Using Ceftazidime-Avibactam as a Therapeutic Option for Bloodstream Infections Caused by Multidrug-Resistant Enterobacterales and Pseudomonas aeruginosa Based on Its Susceptibility Profile

**DOI:** 10.7759/cureus.c236

**Published:** 2025-08-11

**Authors:** Ketan Priyadarshi, Sarumathi Dhandapani, Monika Sivaradjy, Lakshmi Shanmugam, Apurba S Sastry

**Affiliations:** 1 Department of Microbiology, All India Institute of Medical Sciences, Patna, IND; 2 Department of Microbiology, Jawaharlal Institute of Postgraduate Medical Education and Research, Puducherry, IND; 3 Department of Microbiology, Employees' State Insurance Corporation Medical College and Post Graduate Institute of Medical Science and Research, Chennai, IND

The Ethics Statement and Conflict of Interest Disclosures section of this article has been corrected to indicate that this study did involve human subjects or tissue.

